# Multidimensional diagnostic strategies for managing primary aldosteronism with false bilateral AVS due to a duplicated right adrenal vein: A case report

**DOI:** 10.1097/MD.0000000000043520

**Published:** 2025-07-25

**Authors:** Meng Ma, Sikui Shen, Buatikamu Abudukerimu, Wei Xie, Mingxi Zou, Ying Chen, Haoming Tian, Yan Ren, Tao Chen

**Affiliations:** a Department of Endocrinology and Metabolism, Adrenal Center, West China Hospital of Sichuan University, Chengdu, Sichuan Province, PR China; b Department of Radiology, West China Hospital of Sichuan University, Chengdu, Sichuan Province, PR China; c Department of Endocrinology and Metabolism, The People’s Hospital of Kizilsu Kyrgyz Autonomous Prefecture, Atushi, Xinjiang, PR China; d Department of Urology, Institute of Urology (Laboratory of Reconstructive Urology), West China Hospital, Sichuan University, Chengdu, Sichuan Province, PR China.

**Keywords:** ^68^Ga-Pentixafor PET/MR, adrenal venous sampling, case report, duplicated right adrenal veins, primary aldosteronism

## Abstract

**Rationale:**

Primary aldosteronism can be categorized into 2 main subtypes: aldosterone-producing adenoma (APA) and idiopathic hyperaldosteronism. The treatment strategies for these 2 subtypes differ markedly: APA requires surgical removal of the adenoma, whereas idiopathic hyperaldosteronism is managed with pharmacological therapy. Adrenal venous sampling (AVS) is the most widely used technique for diagnosing APA, but its results can be influenced by abnormal adrenal or tumor venous drainage.

**Patient concerns:**

A 58-year-old female was admitted due to elevated blood pressure for over 20 years and persistent hypokalemia for 3 months, accompanied by reduced physical strength, nocturia (2–3 times per night, 200–300 mL each time), and poor sleep quality.

**Diagnoses:**

The AVS results of the patient did not demonstrate lateralized aldosterone secretion. However, the patient’s Kupers and Kobayashi predictive scores, as well as elevated levels of 18-hydroxycortisol and 11-deoxycorticosterone, all supported the presence of a unilateral APA. Retrospective analysis of contrast-enhanced adrenal computed tomography revealed a right adrenal adenoma with a solitary vein draining directly into the inferior vena cava. In addition, ^68^Ga-pentixafor positron emission tomography/magnetic resonance imaging supported the diagnosis of a right-sided APA.

**Interventions:**

Right adrenalectomy was performed.

**Outcomes:**

At 1 and 3 months postoperatively, the patient exhibited complete clinical and biochemical remission.

**Lessons:**

This case highlights that when there is discordance between AVS findings and clinical assessments (e.g., predictive models), a multifaceted approach incorporating predictive scores, 18-hydroxycortisol, 11-deoxycorticosterone levels, and ^68^Ga-pentixafor positron emission tomography/magnetic resonance imaging can facilitate accurate diagnosis and guide management decisions.

## 1. Introduction

Primary aldosteronism (PA) is a clinical syndrome caused by the autonomous overproduction of aldosterone from the zona glomerulosa of the adrenal cortex, primarily manifesting as hypertension, with or without hypokalemia. Epidemiological data suggest that the prevalence of PA among hypertensive patients is at least >5%.^[1]^ Compared to essential hypertension with similar blood pressure levels, patients with PA have a significantly higher risk of atrial fibrillation, myocardial infarction, stroke, and ventricular hypertrophy.^[2]^ The main etiologies of PA include aldosterone-producing adenoma (APA) and idiopathic hyperaldosteronism (IHA). APA can be cured by surgical removal, whereas IHA is typically treated with mineralocorticoid receptor antagonists.

The diagnosis of PA involves screening, confirmation, and subtype classification, with subtype differentiation being particularly important yet challenging. Common methods for subtype diagnosis include computed tomography (CT), bilateral adrenal venous sampling (AVS), functional imaging, and chemokine receptor imaging. All patients with PA should undergo imaging to exclude large adrenal masses or malignancies. Adrenal CT can distinguish adenomas from bilateral hyperplasia but cannot differentiate APA from nonfunctioning adenomas. The overall sensitivity and specificity of CT for PA subtype diagnosis are 78% and 75%, respectively. AVS is currently the most widely used method for subtyping but is limited by cannulation failure, technical challenges, and inconsistent diagnostic criteria. Furthermore, anatomical variations in adrenal vein drainage may lead to misclassification.

The Kupers predictive score can be used to predict APA, with a sensitivity of 53% (95% confidence interval, 38–68) and specificity of 100% (95% confidence interval, 91–100).^[3]^ The Kobayashi score can predict IHA,but with a positive predictive value of 93.5% when the score is at least 8.^[4]^ Additionally, studies have found elevated levels of steroid metabolites, such as 11-deoxycorticosterone, 18-hydroxycorticosterone, and 18-hydroxycortisol in patients with APA, with a sensitivity of 69% and specificity of 94% for diagnosing APA with KCNJ5 gene mutation.^[5]^ In 2018, Heinze et al identified CXC chemokine receptor 4 (CXCR4) expression in APA, and subsequent studies demonstrated that ^68^Ga-Pentixafor PET-CT has potential for distinguishing APA, with a concordance rate of up to 90% with AVS results and a diagnostic accuracy of 92.3%.^[6]^ This imaging technique can provide an effective alternative for patients who are unable to undergo AVS or have inconclusive AVS results.

We report a case in which AVS results did not demonstrate lateralized aldosterone secretion due to dual right adrenal veins. However, the patient’s clinical features, predictive scores, and elevated steroid metabolites all strongly supported the presence of an APA. ^68^Ga-Pentixafor positron emission tomography/magnetic resonance imaging (PET/MR) imaging confirmed the diagnosis of APA, and postoperative pathology showed cytochrome P450 (CYP) CYP11B2 positivity, consistent with an APA. Follow-up showed complete resolution of hypertension and normalization of biochemical parameters, further validating the diagnosis.

## 2. Case presentation

A female in her 50s was admitted due to elevated blood pressure for over 20 years and persistent hypokalemia for 3 months, accompanied by reduced physical strength, nocturia (2–3 times/night, 200–300 mL each time), and poor sleep quality. Physical examination revealed a body temperature of 36.5°C, a heart rate of 72 beats/min, respiratory rate of 20 breaths/min, and BMI of 19.8 kg/m². Blood pressure was measured in 4 limbs, showing right upper limb: 157/100 mm Hg, right lower limb: 165/100 mm Hg, left upper limb: 147/72 mm Hg, and left lower limb: 174/98 mm Hg. No signs of moon facies, plethora, buffalo hump, skin thinning, or purple striae were observed, and other physical findings were unremarkable.

Laboratory evaluations revealed metabolic alkalosis (pH 7.477, PaCO₂ 42.5 mm Hg, standard bicarbonate 29.7 mmol/L, actual bicarbonate 30.7 mmol/L), hypokalemia (2.14 mmol/L), and a random urine potassium-to-creatinine ratio of 22.89. Uric acid was elevated at 398 μmol/L (normal range: 15–357 μmol/L), triglycerides were 1.81 mmol/L (normal range: 0.29–1.7 mmol/L), total cholesterol was 6.83 mmol/L (normal range: 3.8–5.17 mmol/L), and low-density lipoprotein chelesterol was elevated at 4.67 mmol/L (normal range < 3.4 mmol/L). Glycated hemoglobin A1c was 6.1%. Complete blood count, urinalysis, stool analysis, bone metabolism markers, and thyroid function tests were all within normal limits.

Adrenal hormone evaluation revealed a recumbent direct renin concentration (DRC) of <0.50 μIU/mL, with plasma aldosterone concentration (PAC) at 90.00 ng/dL, yielding an aldosterone-to-renin ratio (ARR) of 180. In the upright position, DRC remained <0.50 μIU/mL, and PAC was 91.80 ng/dL, with an ARR of 183.6. Early morning adrenocorticotropic hormone was 28.02 ng/L (normal range: 5–78 ng/L), Early morning cortisol was 372.00 nmol/L (normal range: 133–537 nmol/L), midnight serum cortisol was 38.10 nmol/L, and 24-hour urinary free cortisol was 58.1 μg/24 hours (normal range: 20.3–127.6 μg/24 hours). Catecholamines and their metabolites were within normal limits. The results of both the saline infusion and captopril challenge tests supported the diagnosis of PA (Table 1).

**Table 1:** Renin angiotensin aldosterone system.

	DRC (μIU/mL)	PAC (ng/dL)	F	K^+^
Supine	<0.50	90.0	372.0	2.71
Standing	<0.50	91.8	–	–
SIT_0	<0.50	96.5	262.0	3.5
SIT_4	<0.50	94.0	150.0	3.67
CCT_0	0.66	33.4	152.0	4.07
CCT_2	<0.50	29.40	162.0	4.19

CCT_0 = pre-captopril challenge test, CCT_2 = post-captopril challenge test, DRC = direct renin concentration, F = early morning cortisol, K^+^ = serum potassium, PAC = plasma aldosterone concentration, SIT_0 = pre-saline load test, SIT_4 = post-saline load test.

**Figure 1.**
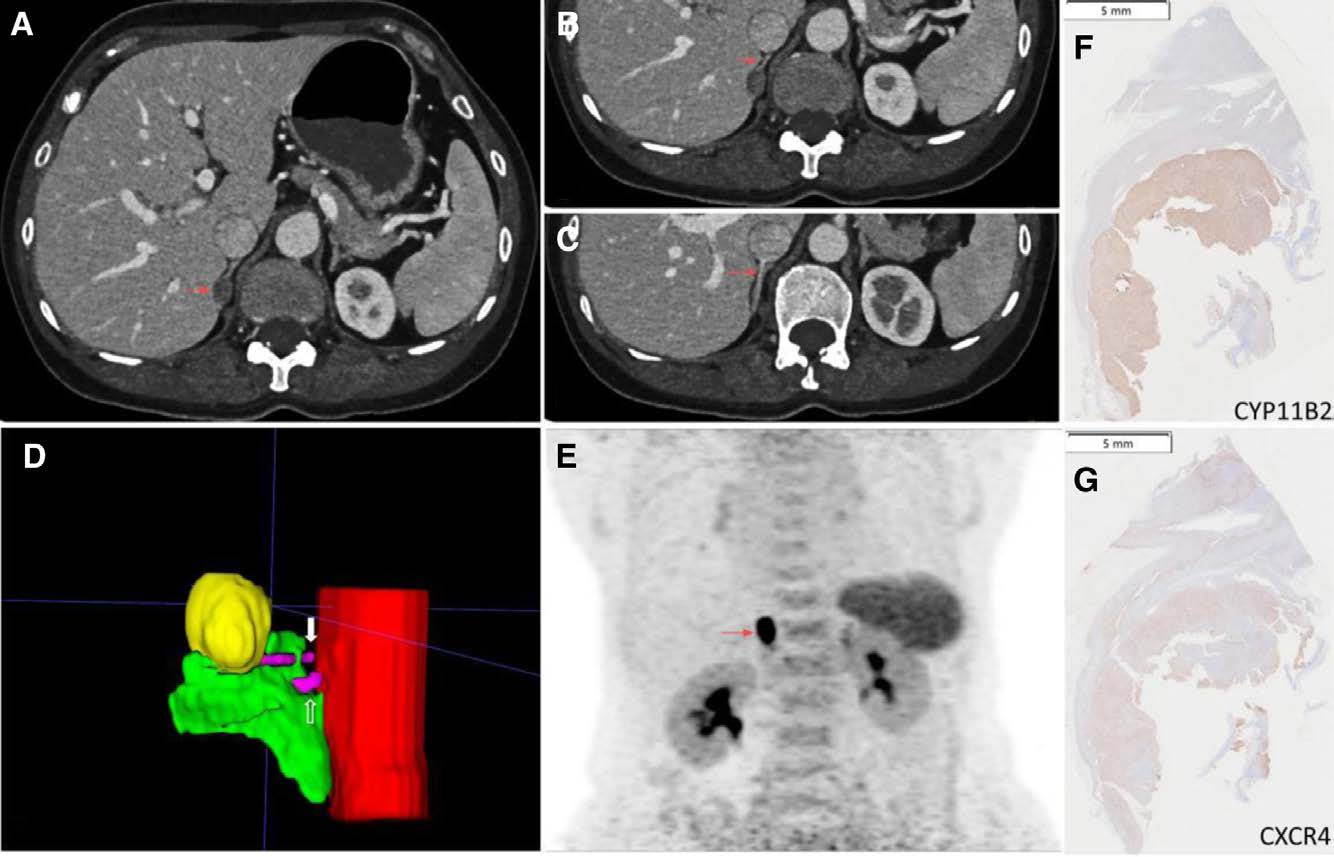
Imaging and IHC results. (A) A 1.3 cm right adrenal adenoma (red arrow) is visible on CT, characterized by an average attenuation of 12 Hounsfield units (HU) and moderate enhancement. (B, C) The upper and lower duplicated right adrenal veins are indicated by red arrows. (D) Schematic representation of a right adrenal adenoma (yellow) within the right adrenal gland (green), with duplicated right adrenal veins – an upper branch (purple,white solid line arrow) and a lower branch (purple, white hollow arrow) – draining into the inferior vena cava (red). (E) ^68^Ga-Pentixafor PET/MR demonstrates significantly increased tracer uptake in the right adrenal nodule (red arrow), with a SUV_max_ 22.85. (F, G) Immunohistochemistry of the resected adenoma shows positive staining for CYP11B2 and CXCR4, confirming its functional status. CT = computed tomography, CXCR4 = CXC chemokine receptor 4, CYP = cytochrome P450, IHC = immunohistochemical analyisis, PET/MR = positron emission tomography/magnetic resonance imaging.

Abdominal contrast-enhanced CT revealed an oval-shaped, low-density nodule in the right adrenal gland, measuring approximately 1.3 cm in diameter, with an average CT value of 12 Hounsfield units (HU) and moderate enhancement, which was suggestive of an adenoma (Fig. 1A). No abnormalities were observed in the left adrenal gland. The Kupers predictive score was 6 (typical Conn adenoma: 3 points, serum potassium level of 2.14 mmol/L: 2 points, estimated glomerular filtration rate of 93.76: 1 point), indicating a diagnosis of APA. The Kobayashi predictive score was 1, indicating a bilateral hyperplasia risk of 15.3%, which did not support bilateral adrenal hyperplasia. The Kocjan score also did not support a diagnosis of IHA.

Mass spectrometry detected significantly elevated levels of steroid metabolites: 11-deoxycorticosterone (167.07 pg/mL; normal range <100 pg/mL), 18-hydroxycorticosterone (1715.25 pg/mL; normal range 90–580 pg/mL), and 18-hydroxycortisol (>4000.00 pg/mL; normal range 198.6–1510.9 pg/mL), consistent with the diagnosis of an APA.

AVS results showed minimal differences in aldosterone levels between both adrenal glands, with a lateralization index (LI) of 1.10, suggesting no significant lateralized aldosterone secretion (Table 2). A retrospective analysis of the contrast-enhanced adrenal CT indicated that the right adrenal nodule’s draining vein emptied directly into the inferior vena cava, which may have contributed to the absence of lateralization in the AVS results (Fig. 1B–D). ^68^Ga-Pentixafor PET/MR revealed significantly increased uptake in the right adrenal nodule (SUV_max_ 22.85), further supporting the diagnosis of APA (Fig. 1E).The patient subsequently underwent laparoscopic right adrenalectomy. During the operation, a yellow, well-encapsulated nodule measuring approximately 1.2 cm in diameter was identified in the right adrenal gland. Postoperative pathology confirmed the presence of a cortical adenoma, and immunohistochemistry demonstrated CYP11B2 positivity, consistent with aldosterone production (Fig. 1F, G). On postoperative day 1, serum potassium increased to 2.81 mmol/L, and blood pressure was 143/88 mm Hg with an ARR of 10.92. At 1 month postoperatively, serum potassium was 5.45 mmol/L, blood pressure ranged between 100–120/70–90 mm Hg, and ARR decreased to 4.12, with PAC reduced by 88.75 ng/dL compared to preoperative levels. At 3 months postoperatively, serum potassium was 4.91 mmol/L, blood pressure was well controlled, ARR was 0.69, and PAC levels had decreased by 88.37 ng/dL, with complete resolution of symptoms.At 6 month postoperatively, serum potassium was 4.89 mmol/L, blood pressure ranged between 120/70–90 mm Hg, and ARR decreased to 1.04, with PAC reduced by 86.44 ng/dL compared to preoperative levels.

**Table 2:** Adrenal venous sampling.

Blood sampling point	Cortisol (nmol/L)	Aldosterone (ng/dL)	Selective index (SI)	Lateralization index (LI)	Corrected aldosterone
Inferior vena cava	814.00	156.20	–	–	0.1919
Left adrenal vein 1	21,985.00	206.00	27.01	1.10	0.0094
Left adrenal vein 2	28,505.00	224.00	35.02	–	0.0079
Right adrenal vein 1	24,217.00	206.00	29.75	–	0.0085

LI = lateralization index, SI = selective index.

## 3. Discussion

This patient presented with long-standing refractory hypertension accompanied by spontaneous hypokalemia, complete suppression of DRC, and significantly elevated PAC (upright aldosterone 90.0 ng/dL). Imaging revealed a right adrenal nodule with features typical of an APA (1.3 cm in diameter, low density, moderate enhancement). Except for age over 40 years, all features were consistent with a clinical diagnosis of APA. The Kupers predictive scores supported the diagnosis of unilateral APA, while the Kobayashi predictive score indicated a low probability of IHA. Steroid metabolite analysis by liquid chromatography-mass spectrometry also revealed significant elevations in 11-deoxycorticosterone, 18-hydroxycorticosterone, and 18-hydroxycortisol, which were consistent with APA. However, the AVS findings indicated no lateralized aldosterone secretion. ^68^Ga- Pentixafor PET/MR revealed a high uptake lesion in the right adrenal gland, and postoperative pathology and CYP11B2 staining confirmed the diagnosis of an APA. Postoperatively, both blood pressure and biochemical parameters were completely normalized.

Accurate subtyping of PA is crucial for selecting appropriate treatment strategies, particularly for distinguishing between unilateral and bilateral disease. Although AVS is the most commonly used technique for subtyping, it has significant limitations, and many factors can result in falsely negative AVS findings, including non-stimulation sampling, stress, hypokalemia, mineralocorticoid receptor antagonists, and cortisol co-secretion.^[7]^ Additionally, published literature has indicated that anatomical abnormalities of the adrenal veins may significantly affect the accuracy of AVS results. For instance, Tannai et al analyzed AVS data from 432 patients with PA and found that 14 patients (3.2%) had dual right adrenal veins, which led to 2 patients having a misclassification of right dominance to no lateralization.^[8]^ Pak J et al also reported a case in which dual right adrenal veins led to inconsistent LI results.^[9]^ Furthermore, there have been reports of duplicated inferior vena cava,^[10]^ left adrenal vein draining directly into the preaortic trunk of the inferior vena cava (left IVC),^[9,11]^ and the right adrenal venous drainage into the right accessory hepatic vein.^[9]^ Vonend et al reported a case of an anatomical variation in the right adrenal vein, which led to misinterpretation of AVS results as bilateral secretion, but after the surgical removal of the right adenoma, the patient’s symptoms were completely resolved. These findings suggest that adrenal venous abnormalities may be an important factor that interferes with AVS results.

In this case, the patient underwent AVS with adrenocorticotropic hormone stimulation, and the sampling indices indicated successful bilateral AVS (SI values of 27.01/35.02 for the left adrenal vein and 29.75 for the right adrenal vein). However, the LI showed no significant aldosterone lateralization (LI 1.10). In addition to AVS, noninvasive predictive models and intermediate steroid metabolites were also used for subtype prediction in PA. The Kupers score primarily predicts unilateral APA, and a high score indicates potential benefit from surgery. This patient’s Kupers score was 6 (maximum score of 7), supporting the diagnosis of APA. The Kobayashi score, primarily used for predicting bilateral PA, helps provide an initial determination of subtype diagnosis. A higher Kobayashi score indicates a greater likelihood of bilateral PA (maximum score of 12, with bilateral PA risk >93.5%). This patient had a Kobayashi score of 1, which did not support bilateral PA. Furthermore, studies have shown that 18-hydroxycorticosterone and 18-hydroxycortisol levels are significantly higher in patients with unilateral APA compared to those with bilateral hyperplasia. In this case, 11-deoxycorticosterone was 167.07 pg/mL (normal range <100 pg/mL), 18-hydroxycorticosterone was 1715.25 pg/mL (normal range 90–580 pg/mL), and 18-hydroxycortisol was >4000.00 pg/mL (normal range 198.6–1510.9 pg/mL), all of which were significantly elevated, suggesting a high likelihood of APA.

Based on the above analysis, we considered that the AVS results in this patient might be a false negative for lateralization. The patient did not experience significant stress (emotional tension, pain, etc) during sampling, hypokalemia had been corrected, and there was no evidence of cortisol co-secretion or use of MRAs that might significantly affect aldosterone secretion. We then reexamined the adrenal CT scan venous phase images, which showed that the patients had dual right adrenal veins (the main upper 1 and the accessory lower 1), and the draining vein of APA might be the lower accessory 1 while adrenal sampling might from the upper main 1, which might have caused the false negative AVS result. To validate this hypothesis, we recommended a ^68^Ga-Pentixafor PET/MR scan. In 2018, Heinze et al first reported CXCR4 expression in aldosterone-secreting cells, with significantly higher expression in APA than in IHA. ^68^Ga-labeled Pentixafor specifically binds to CXCR4, allowing visualization of APA.^[6]^ Subsequent studies indicated that the sensitivity, specificity, and accuracy of ^68^Ga-Pentixafor positron emission tomography/computed tomography (PET/CT) for diagnosing APA were 100%, 78.6%, and 92.3%, respectively (SUV 11.18), with a concordance rate of up to 90% between ^68^Ga-Pentixafor PET/CT and AVS.^[12]^ In this case, ^68^Ga-Pentixafor PET/MR showed an SUV_max_ of 22.85 for the right adrenal nodule, while the rest of the right adrenal cortex and the left adrenal cortex had SUV_max_ values of 4.23 and 4.69, respectively, supporting the diagnosis of right APA. After thorough discussion with the patient, right adrenalectomy was performed, and immunohistochemistry showed CYP11B2 positivity, consistent with APA. Follow-up at 6 months postoperatively showed normalization of blood pressure, potassium levels, DRC, and PAC.

The diagnosis and treatment journey of this case emphasize the importance of integrating multiple diagnostic methods, particularly when AVS findings do not align with clinical features, and anatomical variations are suspected. Detailed interpretation of CT images and novel imaging modalities such as ^68^Ga-Pentixafor PET/CT can significantly improve diagnostic accuracy. Additionally, this case reminds clinicians to be cautious when interpreting AVS results, particularly in the presence of adrenal vein anatomical anomalies. A comprehensive evaluation using predictive models, steroid metabolite profiling, and advanced imaging can ensure a more accurate diagnosis and treatment plan for patients.

This case still has certain limitations: firstly, due to the retrospective nature of the study, detailed observations and documentation of venous anomalies during AVS could not be performed. Secondly, the exact anatomical evidence of tumor draining veins into the inferior vena cava was not clearly established intraoperatively.

In conclusion, this case describes a patient with PA in whom an anomalous tumor draining vein led to a false negative AVS result. It suggests that, when clinical assessment and AVS findings are inconsistent, the possibility of a false negative AVS result should be considered. A multi-angle analysis using predictive models, steroid metabolite profiling, and novel imaging modalities can help ensure diagnostic and therapeutic accuracy.

## Acknowledgments

The study was supported by a Grant from the Science & Technology Department of Sichuan Province (23ZDYF2910) and a Grant from the Ministry of Science and Technology of the People’s Republic of China (2022YFC2505300).

## Author contributions

**Conceptualization:** Tao Chen.

**Data curation:** Meng Ma.

**Formal analysis:** Meng Ma.

**Investigation:** Meng Ma, Sikui Shen, Buatikamu Abudukerimu.

**Project administration:** Haoming Tian, Yan Ren.

**Validation:** Sikui Shen.

**Writing** – **original draft:** Meng Ma.

**Writing** – **review & editing:** Sikui Shen, Buatikamu Abudukerimu, Wei Xie, Mingxi Zou, Ying Chen, Haoming Tian, Tao Chen, Yan Ren.
